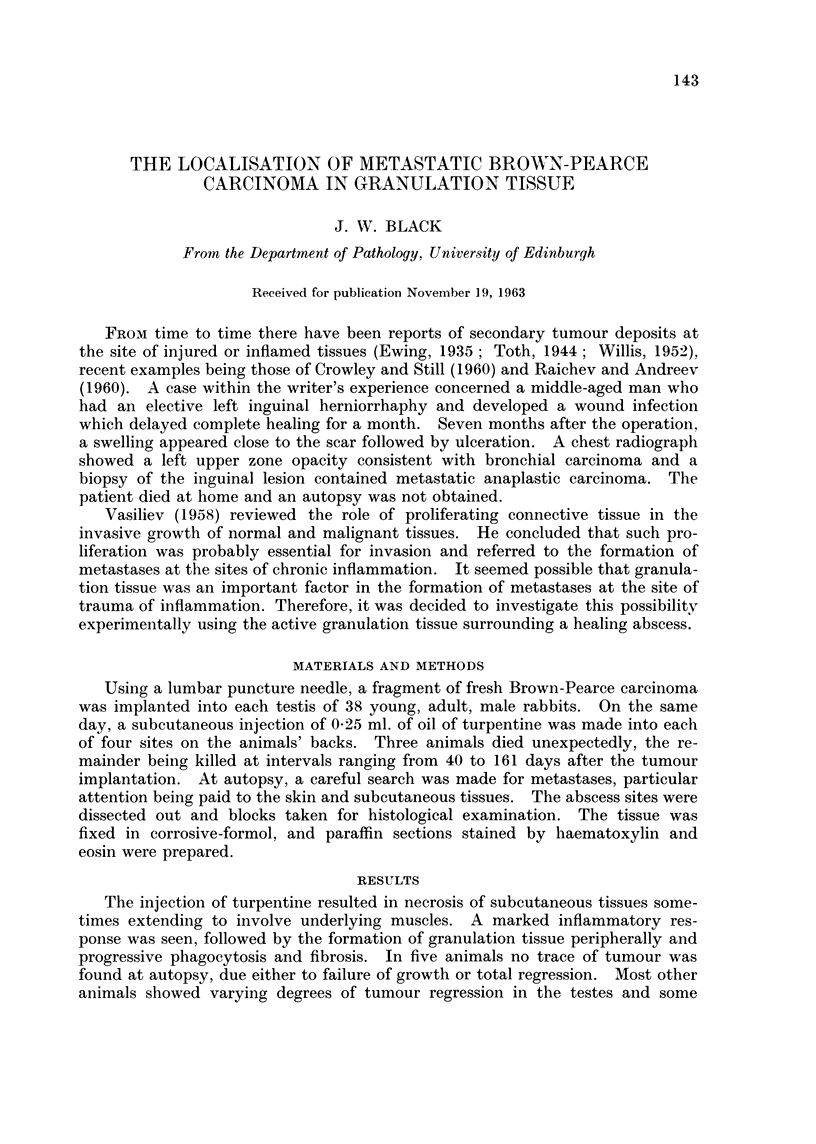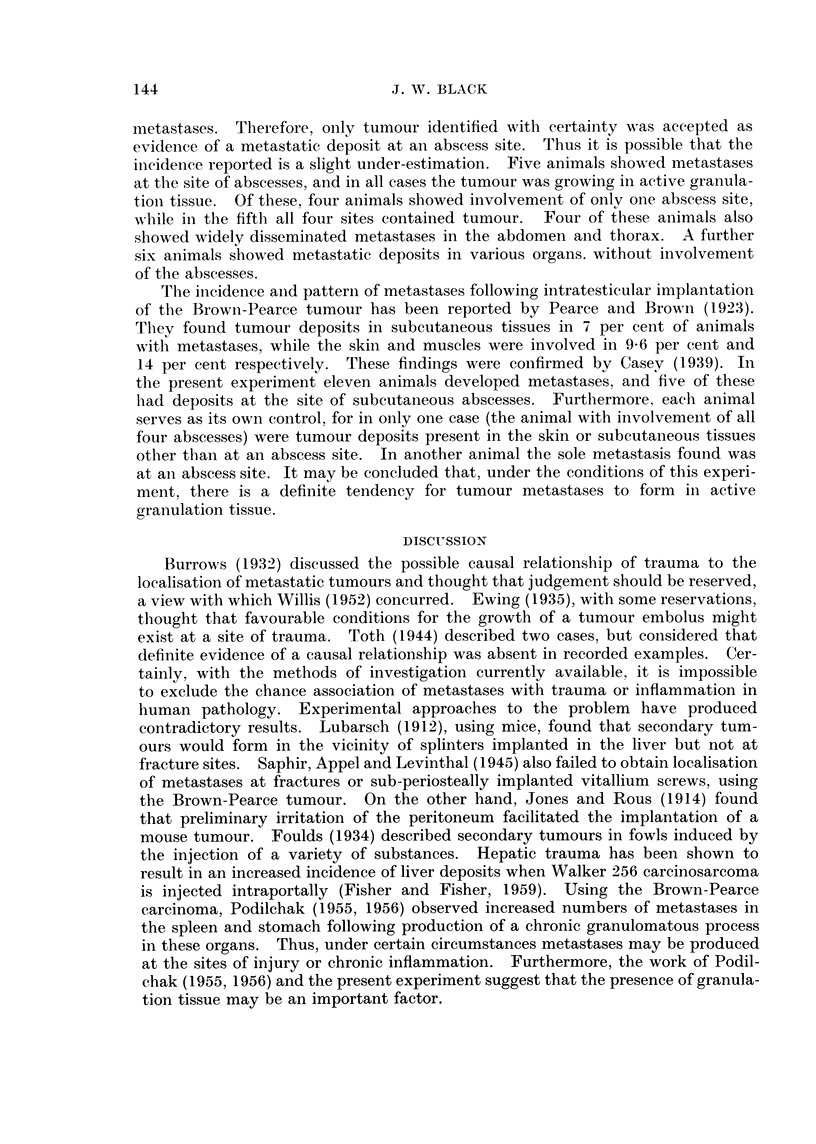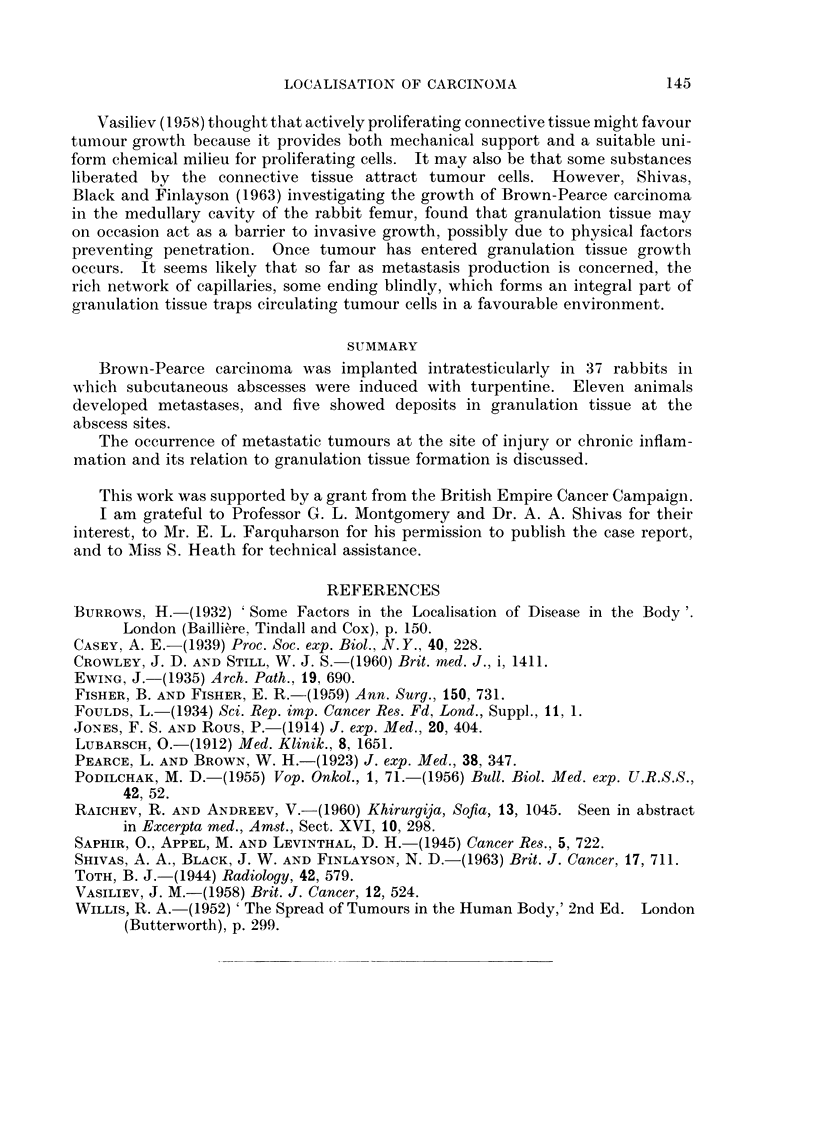# The Localisation of Metastatic Brown-Pearce Carcinoma in Granulation Tissue

**DOI:** 10.1038/bjc.1964.15

**Published:** 1964-03

**Authors:** J. W. Black


### THE LOCALISATION OF METASTATIC BROWN-PEARCE CARCINOMA IN GRANULATION TISSUE

J. W. BLACK

*From the Department of Pathology, University of Edinburgh*

Received for publication November 19, 1963

FROM time to time there have been reports of secondary tumour deposits at the site of injured or inflamed tissues (Ewing, 1935 ; Toth, 1944 ; Willis, 1952), recent examples being those of Crowley and Still (1960) and Raichev and Andreev (1960). A case within the writer's experience concerned a middle-aged man who had an elective left inguinal herniorrhaphy and developed a wound infection which delayed complete healing for a month. Seven months after the operation, a swelling appeared close to the scar followed by ulceration. A chest radiograph showed a left upper zone opacity consistent with bronchial carcinoma and a biopsy of the inguinal lesion contained metastatic anaplastic carcinoma. The patient died at home and an autopsy was not obtained.

Vasiliev (1958) reviewed the role of proliferating connective tissue in the invasive growth of normal and malignant tissues. He concluded that such proliferation was probably essential for invasion and referred to the formation of metastases at the sites of chronic inflammation. It seemed possible that granulation tissue was an important factor in the formation of metastases at the site of trauma of inflammation. Therefore, it was decided to investigate this possibility experimentally using the active granulation tissue surrounding a healing abscess.

### MATERIALS AND METHODS

Using a lumbar puncture needle, a fragment of fresh Brown-Pearce carcinoma was implanted into each testis of 38 young, adult, male rabbits. On the same day, a subcutaneous injection of 0·25 ml. of oil of turpentine was made into each of four sites on the animals' backs. Three animals died unexpectedly, the remainder being killed at intervals ranging from 40 to 161 days after the tumour implantation. At autopsy, a careful search was made for metastases, particular attention being paid to the skin and subcutaneous tissues. The abscess sites were dissected out and blocks taken for histological examination. The tissue was fixed in corrosive-formol, and paraffin sections stained by haematoxylin and eosin were prepared.

### RESULTS

The injection of turpentine resulted in necrosis of subcutaneous tissues sometimes extending to involve underlying muscles. A marked inflammatory response was seen, followed by the formation of granulation tissue peripherally and progressive phagocytosis and fibrosis. In five animals no trace of tumour was found at autopsy, due either to failure of growth or total regression. Most other animals showed varying degrees of tumour regression in the testes and some metastases. Therefore, only tumour identified with certainty was accepted as evidence of a metastatic deposit at an abscess site. Thus it is possible that the incidence reported is a slight under-estimation. Five animals showed metastases at the site of abscesses, and in all cases the tumour was growing in active granulation tissue. Of these, four animals showed involvement of only one abscess site, while in the fifth all four sites contained tumour. Four of these animals also showed widely disseminated metastases in the abdomen and thorax. A further six animals showed metastatic deposits in various organs. without involvement of the abscesses.

The incidence and pattern of metastases following intratesticular implantation of the Brown-Pearce tumour has been reported by Pearce and Brown (1923). They found tumour deposits in subcutaneous tissues in 7 per cent of animals with metastases, while the skin and muscles were involved in 9·6 per cent and 14 per cent respectively. These findings were confirmed by Casey (1939). In the present experiment eleven animals developed metastases, and five of these had deposits at the site of subcutaneous abscesses. Furthermore, each animal serves as its own control, for in only one case (the animal with involvement of all four abscesses) were tumour deposits present in the skin or subcutaneous tissues other than at an abscess site. In another animal the sole metastasis found was at an abscess site. It may be concluded that, under the conditions of this experiment, there is a definite tendency for tumour metastases to form in active granulation tissue.

### DISCUSSION

Burrows (1932) discussed the possible causal relationship of trauma to the localisation of metastatic tumours and thought that judgement should be reserved, a view with which Willis (1952) concurred. Ewing (1935), with some reservations, thought that favourable conditions for the growth of a tumour embolus might exist at a site of trauma. Toth (1944) described two cases, but considered that definite evidence of a causal relationship was absent in recorded examples. Certainly, with the methods of investigation currently available, it is impossible to exclude the chance association of metastases with trauma or inflammation in human pathology. Experimental approaches to the problem have produced contradictory results. Lubarsch (1912), using mice, found that secondary tumours would form in the vicinity of splinters implanted in the liver but not at fracture sites. Saphir, Appel and Levinthal (1945) also failed to obtain localisation of metastases at fractures or sub-periosteally implanted vitallium screws, using the Brown-Pearce tumour. On the other hand, Jones and Rous (1914) found that preliminary irritation of the peritoneum facilitated the implantation of a mouse tumour. Foulds (1934) described secondary tumours in fowls induced by the injection of a variety of substances. Hepatic trauma has been shown to result in an increased incidence of liver deposits when Walker 256 carcinosarcoma is injected intraportally (Fisher and Fisher, 1959). Using the Brown-Pearce carcinoma, Podilchak (1955, 1956) observed increased numbers of metastases in the spleen and stomach following production of a chronic granulomatous process in these organs. Thus, under certain circumstances metastases may be produced at the sites of injury or chronic inflammation. Furthermore, the work of Podilchak (1955, 1956) and the present experiment suggest that the presence of granulation tissue may be an important factor.

Vasiliev (1958) thought that actively proliferating connective tissue might favour tumour growth because it provides both mechanical support and a suitable uniform chemical milieu for proliferating cells. It may also be that some substances liberated by the connective tissue attract tumour cells. However, Shivas, Black and Finlayson (1963) investigating the growth of Brown-Pearce carcinoma in the medullary cavity of the rabbit femur, found that granulation tissue may on occasion act as a barrier to invasive growth, possibly due to physical factors preventing penetration. Once tumour has entered granulation tissue growth occurs. It seems likely that so far as metastasis production is concerned, the rich network of capillaries, some ending blindly, which forms an integral part of granulation tissue traps circulating tumour cells in a favourable environment.

### SUMMARY

Brown-Pearce carcinoma was implanted intratesticularly in 37 rabbits in which subcutaneous abscesses were induced with turpentine. Eleven animals developed metastases, and five showed deposits in granulation tissue at the abscess sites.

The occurrence of metastatic tumours at the site of injury or chronic inflammation and its relation to granulation tissue formation is discussed.

This work was supported by a grant from the British Empire Cancer Campaign. I am grateful to Professor G. L. Montgomery and Dr. A. A. Shivas for their interest, to Mr. E. L. Farquharson for his permission to publish the case report, and to Miss S. Heath for technical assistance.

### REFERENCES

Burrows, H.—(1932) ' Some Factors in the Localisation of Disease in the Body '. London (Baillière, Tindall and Cox), p. 150.

Casey, A. E.—(1939) *Proc. Soc. exp. Biol., N.Y.*, **40**, 228.

Crowley, J. D. and Still, W. J. S.—(1960) *Brit. med. J.*, i, 1411.

Ewing, J.—(1935) *Arch. Path.*, **19**, 690.

Fisher, B. and Fisher, E. R.—(1959) *Ann. Surg.*, **150**, 731.

Foulds, L.—(1934) *Sci. Rep. imp. Cancer Res. Fd, Lond.*, Suppl., **11**, 1.

Jones, F. S. and Rous, P.—(1914) *J. exp. Med.*, **20**, 404.

Lubarsch, O.—(1912) *Med. Klinik.*, **8**, 1651.

Pearce, L. and Brown, W. H.—(1923) *J. exp. Med.*, **38**, 347.

Podilchak, M. D.—(1955) *Vop. Onkol.*, **1**, 71.—(1956) *Bull. Biol. Med. exp. U.R.S.S.*, **42**, 52.

Raichev, R. and Andreev, V.—(1960) *Khirurgija, Sofia*, **13**, 1045. Seen in abstract in *Excerpta med., Amst.*, Sect. XVI, **10**, 298.

Saphir, O., Appel, M. and Levinthal, D. H.—(1945) *Cancer Res.*, **5**, 722.

Shivas, A. A., Black, J. W. and Finlayson, N. D.—(1963) *Brit. J. Cancer*, **17**, 711.

Toth, B. J.—(1944) *Radiology*, **42**, 579.

Vasiliev, J. M.—(1958) *Brit. J. Cancer*, **12**, 524.

Willis, R. A.—(1952) ' The Spread of Tumours in the Human Body,' 2nd Ed. London (Butterworth), p. 299.